# Predictors of the Prevalence of Dyslipidemia and Influencing Factors for Young Health Examination Cohort: A Cross-Sectional Survey

**DOI:** 10.3389/fpubh.2020.00400

**Published:** 2020-09-23

**Authors:** Hui Zhang, William Robert Kwapong, Meng-Meng Shao, Jue-Yue Yan, Xian-Da Lin, Bo-Bei Chen, Ke-Yang Chen

**Affiliations:** ^1^Department of Pediatric Allergy and Immunology, The Second Affiliated Hospital and Yuying Children's Hospital of Wenzhou Medical University, Wenzhou, China; ^2^School of Ophthalmology and Optometry, Wenzhou Medical University, Wenzhou, China; ^3^Department of Rehabilitation, The First Affiliated Hospital of Wenzhou Medical University, Wenzhou, China; ^4^Department of Neurology, The Second Affiliated Hospital and Yuying Children's Hospital of Wenzhou Medical University, Wenzhou, China; ^5^Department of Neurology, Wenzhou Peoples' Hospital, Wenzhou, China; ^6^Department of Otolaryngology, The Second Affiliated Hospital and Yuying Children's Hospital of Wenzhou Medical University, Wenzhou, China

**Keywords:** dyslipidemia, prevalence, associated risk factors, health examination, young population

## Abstract

**Objectives:** The objective of this study was to estimate the prevalence of dyslipidemia and associated influencing factors in young adults in the southeastern coastal area of China.

**Methods:** This study adopted a cross-sectional survey and included 7,859 young people who underwent examinations at three hospitals in Wenzhou, Zhejiang Province, China. All subjects completed a questionnaire in the form of face-to-face interviews and underwent anthropometric measurements and biochemical tests. The continuous data are presented as the means ± standard deviations and were compared using Student's *t*-tests. The categorical variables are presented as proportions. The influencing factors associated with dyslipidemia were evaluated through a multivariate logistic regression.

**Results:** The prevalence of dyslipidemia among young adults aged 18–45 years in the southeastern coast of China was high with 7.1, 15.0, 22.9, and 4.0% for high-total cholesterol (TC), high-triglyceride (TG), low-high-density lipoprotein cholesterol (HDL-C), and high-low-density lipoprotein cholesterol (LDL-C). Among those with dyslipidemia, a statistically significant difference in sex was observed, and all types of dyslipidemia were associated with smoking and alcohol consumption. However, those with high-TG, high-LDL, and low-HDL levels did not significantly differ in education level or occupation. The presence of dyslipidemia was significantly associated with increased age, the male sex (OR: 1.85, 95% CI: 1.39–2.21), smoking (OR: 2.02, 95% CI: 1.98–2.13), alcohol consumption (OR: 1.33, 95% CI: 1.16–1.63), overweight or obesity (OR: 2.01, 95% CI: 1.79–2.41), and intellectual work (OR: 1.36, 95% CI: 1.11–1.72).

**Conclusion:** The prevalence of dyslipidemia among young adults aged 18–45 years in the southeastern coast of China was high. To prevent dyslipidemia at an early age, it is essential to conduct effective intervention programs targeting risk factors and to implement routine screening programs.

## Introduction

The prevalence of stroke is increasing and stroke is reportedly among the most common causes of mortality and morbidity worldwide (1). In China, it has been reported that the prevalence of stroke in the elderly is very high (2). A recent report has shown that the global burden of stroke is alarming. Thus, researchers and clinicians are exploring the risk factors for stroke among youth to assist with prevention (3). Stroke is common but rare in young people (age range of 18–45) (4). However, a report showed a gradual increase in younger persons (5). The increasing incidence of stroke in young people has been attributed to the increasing trends of multiple traditional risk factors including dyslipidemia, obesity, diabetes, and hypertension (6).

Dyslipidemia refers to abnormal levels of lipids in the blood and is usually represented as elevated total cholesterol (TC), triglycerides (TGs), and/or low- density lipoprotein cholesterol (LDL), and a decrease in high-density lipoprotein cholesterol (HDL) (7). Dyslipidemia is a major risk factor for cardiovascular and cerebrovascular diseases. The first national lipid study was the China National Nutrition and Health Survey launched in 2002 by China's Center for Disease Control and Prevention. The survey revealed that the prevalence of dyslipidemia in Chinese adults aged 18 and older is 18.6% (8). Due to changes in China's economy and lifestyle, dyslipidemia has become a prevalent health issue in younger people in recent years. However, very limited studies investigating dyslipidemia have been conducted among young people in China, especially in the southeastern coastal area. It is critical to collect more epidemiological data regarding dyslipidemia and cerebrovascular risk factors to improve early screening and effective lipid level management. We aimed to compile useful information regarding the health of people and create a database of data from a young population by performing medical examinations that could be useful to the local government health management department in substantially reducing the burden of stroke and providing social and economic value in this area.

## Methods

### Study Design

An observational cross-sectional survey targeting young adults was conducted in three hospitals in Wenzhou, Zhejiang Province. We obtained informed consent from all participants. The patients participated in this study voluntarily and were informed of the purpose of the study and their rights through the consent form. This study was approved by the Ethics Committee of The Second Affiliated Hospital of Wenzhou Medical University.

### Sample

The data were collected from a young population comprising of individuals aged 18–45 years, who visited any of the three hospitals for a medical health examination from April 2017 to December 2018. Each hospital's health examination center randomly selected all individuals. The subjects were selected through a two-stage random sampling procedure. During the first stage of sampling, three tertiary A hospitals were selected from eight tertiary A hospitals using a computer-generated random number table. Each participant had a social security card and physical examination record. During the second stage of sampling, the participants were selected for a physical examination using another computer-generated random number table in each hospital, resulting in a total of 8,703 patients in the young health examination cohort, with 682 of them refusing to contribute. We excluded participants who were too mentally disabled to cooperate with the investigator; were pregnant; had a history of cardiovascular and cerebrovascular diseases, renal insufficiency; or had a history of using steroids, statins, contraceptive pills, or other medicines possibly related to dyslipidemia. Finally, in total, 7,859 participants were enrolled. The response rate was ~90.3%.

### Data Collection

The data were collected by investigators through face-to-face interviews. All researchers underwent training related to the research objectives, measurement methods, the importance of standardization, and the method used to complete the questionnaire. The investigators used standardized questionnaires designed by professionals to collect the data, including demographic information (age, sex, and level of education) health behaviors (alcohol consumption, smoking, and physical activity), and history of chronic diseases (hypertension and diabetes). The body mass index (BMI) was calculated as weight in kilograms divided by the square of height in meters. Blood pressure readings were taken from the participants' right arms using a standardized automatic electronic sphygmomanometer. The measurements were performed three times at 2-min intervals. The systolic blood pressure (SBP) and diastolic blood pressure (DBP) were recorded.

### Laboratory Measurements

Blood samples were obtained in the morning. The biochemical parameters were measured at the laboratory of The Second Affiliated Hospital and Yuying Children's Hospital of Wenzhou Medical University. All control values were consistent with the standards recommended by the medical laboratory of the China Center for Disease Control and Prevention. All laboratory technicians were trained in formal laboratory biosafety and biosecurity procedures. The TC, TGs, HDL-C, blood uric acid (UA), and fasting plasma glucose (FPG) levels were all measured. The concentration of LDL-C was calculated using the Friedewald formula (9).

### Variables

Dyslipidemia was defined according to the Chinese guidelines for the prevention and treatment of dyslipidemia in adults as follows (10): TGs ≥ 2.26 mmol/L (200 mg/dL); TC ≥ 6.22 mmol/L(240 mg/dL); LDL-C ≥ 4.14 mmol/L(160 mg/dL); and HDL-C <1.04 mmol/L (40 mg/dL). Hypertension was defined as an SBP ≥ 140 mmHg and/or a DBP ≥ 90 mmHg or self-reported treatment with hypertensive medication. Overweight and obesity were defined as a BMI ≥ 24 and <28 and ≥28 kg/m^2^, according to the cut-off points for Chinese adults (11). Diabetes mellitus was diagnosed using the following WHO criteria: FPG ≥ 7 mmol/L (126 mg/dL). Smoking status was categorized according to 1 year of smoking at least one cigarette per day (12). Alcohol consumption was considered in terms of whether the participant consumed alcohol at least 12 times in 1 year. Physical activity per day was defined by classifying exercise time into the following three levels: <30 min as level 1, 30 min^−1^ h as level 2, and more than 1 h as level 3. Occupation types were categorized as follows: intellectual workers included all types of technical personnel, state workers, heads of enterprises and institutions, clerks, and students; manual workers included commercial workers, service workers, agriculture workers, forestry workers, animal husbandry workers, fishermen, and transportation workers; and others included unemployed workers. Education level was categorized as primary school, middle school, high school, or university and higher.

### Analysis

EpiData software V.3.1 was used for double data entry. The means and standard deviations of the continuous variables were provided, and the prevalence and corresponding 95% confidence intervals (CIs) of the categorical variables were calculated. Student's *t*-test was used to test the differences in the means of the continuous variables, and the chi-square test was used to test the differences in the categorical variables. The analyses were sex specific. A multivariable logistic regression analysis, performed using a backward elimination method, was used to determine the factors independently associated with dyslipidemia in young adults. *P*-values < 0.05 were considered statistically significant.

## Results

### Baseline Characteristics of the Study Population

**Figure 1:**
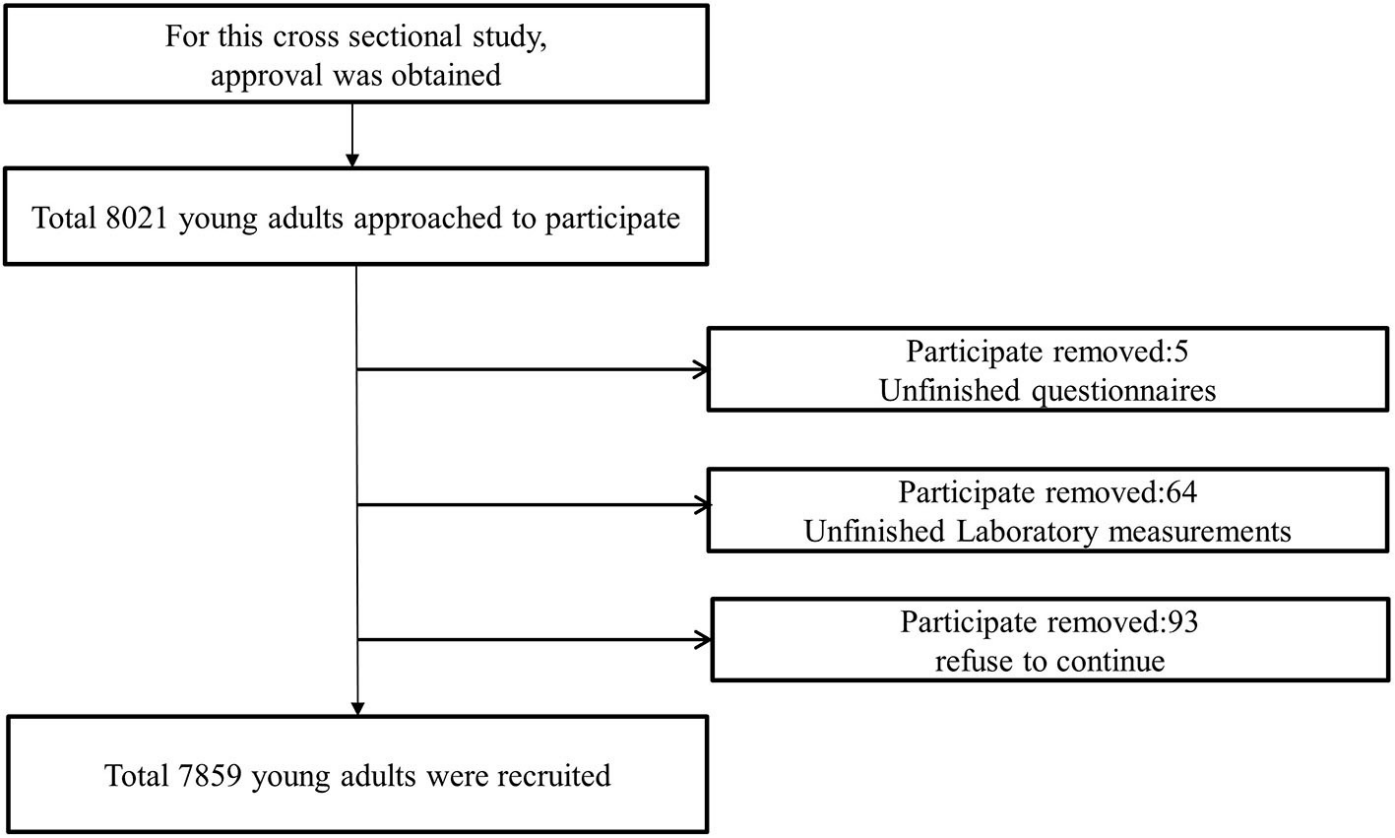
Flow chart of sample inclusion and exclusion.

**Figure 2:**
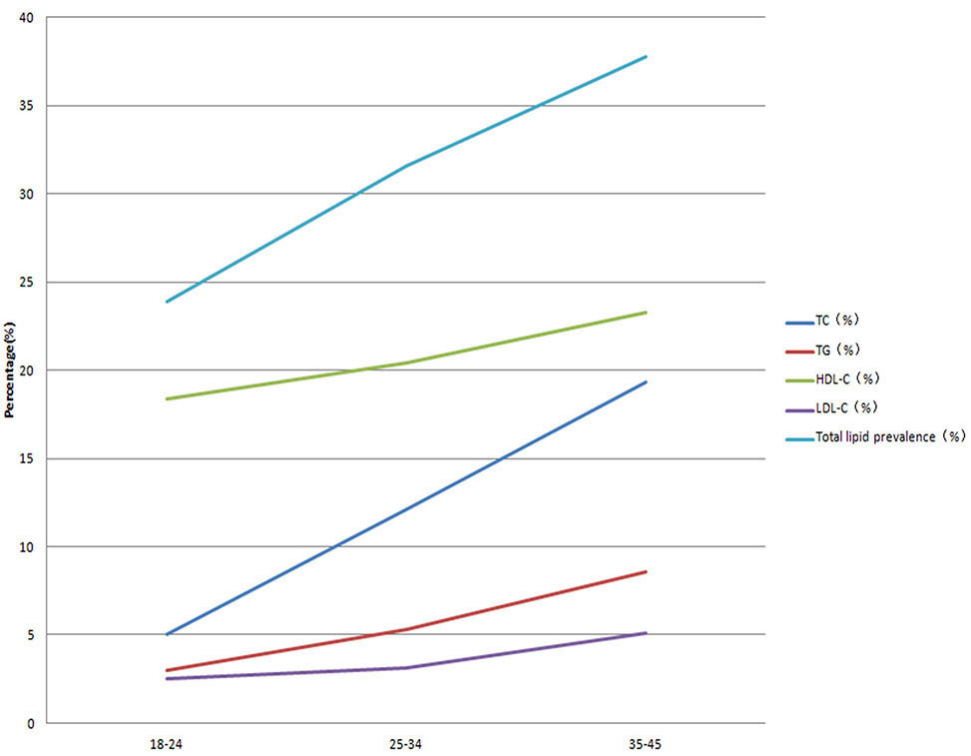
Prevalence of dyslipidemia by different ages.

In total, 7,859 participants were recruited for this study (Figure 1). Table 1 shows the baseline characteristics of the participants. The study participants included 3,968 males (50.5%) with an average age of 34.07 ± 7.11 years and 3,891 females (49.5%), with an average age of 34.52 ± 7.08 years. The number of urban participants was 3,827 (48.7%), and that of rural participants was 4,032 (51.3%). The BMI of the men and women were 23.18 ± 2.58 and 22.53 ± 3.85 kg/m^2^, respectively. The levels of glucose and uric acid were 5.03 ± 1.59 mmol/L and 314.76 ± 83.91 μmol/L in the men and 5.24 ± 1.27 mmol/L and 271.91 ± 65.40 μmol/L in the women, respectively. The serum uric acid level, SBP, and DBP in the males were significantly greater than those in the females, and no difference in BMI or glucose levels was observed between the sexes. Smoking and alcohol consumption were more frequent in the men than in the women. The prevalence of dyslipidemia in TC, TGs, HDL-C, and LDL-C increased with age (Figure 2). There were no differences in occupation, physical activity, or education level between the two sexes.

**Table 1:** Demographic, plasma biochemical characteristics of young adults.

**Characteristics**		**Male (*N* = 3,968)**	**Female (*N* = 3,891)**	
Clinic variables	Age (years)		34.07 ± 7.11	34.52 ± 7.08
	Residence (*n*)	Urban	1,933	1,894
		Rural	2,035	1,997
	BMI (kg/m^2^)		23.18 ± 2.58	22.53 ± 3.85
	SBP (mmHg)		120.1	114.2^*^
	DBP (mmHg)		79.3	73.2^*^
Biochemical variables	TC (mmol/L)		5.02 ± 0.96	4.76 ± 0.83^*^
	TG (mmol/L)		1.94 ± 0.64	1.10 ± 0.69^*^
	LDL-C(mmol/L)		2.88 ± 0.81	2.55 ± 0.70^*^
	HDL-C(mmol/L)		1.16 ± 0.27	1.25 ± 0.34^*^
	Glucose(mmol/L)		5.03 ± 1.59	5.14 ± 1.27
	UA (μmol/L)		314.76 ± 83.91	271.91 ± 65.40^*^
Lifestyle variables	Smoking		24.8%	1.28%^*^
	Drinking		18.1%	7.04%^*^
	Physical activity	Level 1	12.8%	13.7%
		Level 2	29.6%	31.2%
		Level 3	57.6%	55.1%
	Occupation	Manual work	51.1%	40.4%
		Intellectual work	48.9%	59.6%
	Education level	Primary	8.6%	9.1%
		Secondary	12.4%	11.2%
		High school	17.7%	16.1%
		University	61.3%	63.6%
	Hypertension		7.9%	3.1%
	Diabetes mellitus		5.8%	6.4%

*BMI, body mass index; SBP, systolic blood pressure; DBP, diastolic blood pressure; TC, Total Cholesterol; TG, triglyceride; LDL-c, low-density lipoprotein cholesterol; HDL-C, high-density lipoprotein cholesterol; UA, uric acid*;

* *P < 0.05, male vs. female*.

### Prevalence of Different Types of Dyslipidemia

**Figure 3:**
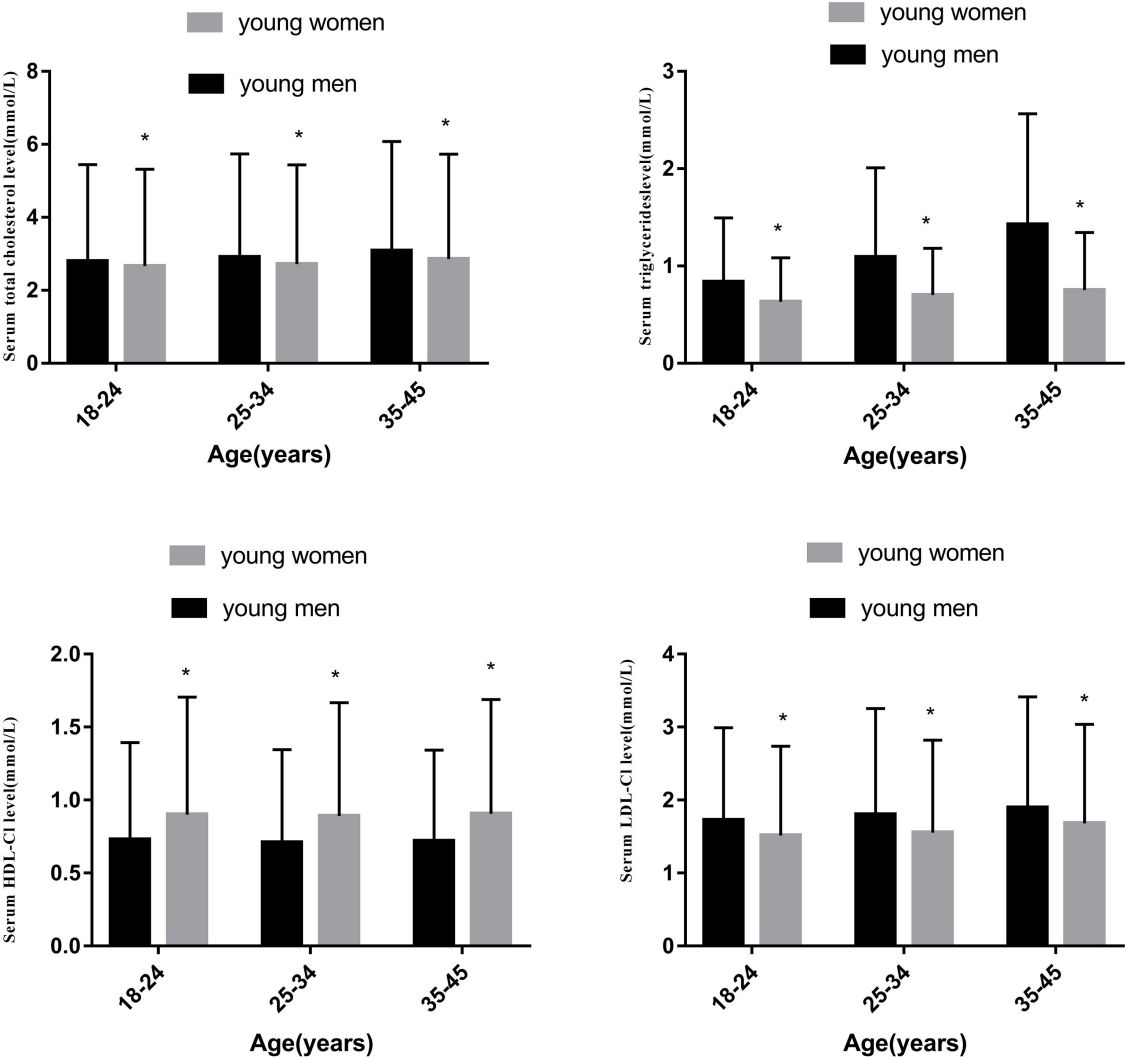
Comparison of two sexes of the concentrations serum in TC, TGs, HDL-C and LDL-C in different age groups.

The overall prevalence of dyslipidemia was 34.11%. Dyslipidemia was more prevalent in the males than females (51.0 vs. 16.7%, *P* = 0.011). The levels of TC, TGs, HDL-C, and LDL-C in the young males were 5.02 ± 0.96, 1.94 ± 0.64, 1.16 ± 0.27, and 2.88 ± 0.81 mmol/L, respectively, and those in the young females were 4.76 ± 0.83, 1.10 ± 0.69, 1.25 ± 0.34, and 2.55 ± 0.70 mmol/L, respectively. Figure 3 suggested that the levels of TC, TGs, LDL-C were higher in the young men, whereas the HDL-C level was lower compared to the young women. The prevalence of high TC, high TG, low HDL-C, and high LDL-C levels was 7.1, 15.0, 22.9, and 4.0%, respectively. As shown in Table 2, the levels of TC, TGs, and LDL-C in the young males and females increased with age (all *P* < 0.05), and the difference in the HDL-C levels by age was not statistically significant (all *P* = 0.094). The prevalence of dyslipidemia significantly differed between the males and females (all *P* < 0.001). Compared with young adults in rural areas, those in urban areas had a similar prevalence of dyslipidemia (all *P* > 0.05). Regarding BMI, the prevalence rates of all types of dyslipidemia among those who were overweight were significantly higher than the rates among those who were not overweight. The prevalence of all types of dyslipidemia was significantly associated with the smoking and alcohol consumption status (all *P* < 0.05). The prevalence of high TC levels decreased with education level (*P* = 0.001). However, the high TG, high LDL, and low HDL levels did not significantly differ according to education level (all *P* > 0.05). The incidence of dyslipidemia did not significantly differ according to occupation (all *P* > 0.05).

**Table 2:** Prevalence of different types of dyslipidemia in young adults (%).

**Category**	**Subcategory**	**H-TC**	**H-TG**	**L-HDL**	**H-LDL**
Age	18–24	5	3	19.4	2.5
	25–34	12.1	5.3	20.4	3.1
	35–45	19.3	8.6	23.3	6.1
	*p*-value	<0.001	<0.001	0.094	<0.001
Gender	Male	9.7	25.1	35.8	5.9
	Female	4.5	4.7	9.3	2.2
	*p*-value	<0.001	<0.001	<0.001	<0.001
Residence	Urban	7.1	15.1	22.3	4.2
	Rural	6.9	15.6	23.5	3.9
	*p*-value	0.861	0.785	0.487	0.835
Physical activity	Level 1	8.1	7.1	11.8	9.5
	Level 2	5.9	5.6	9.1	3.4
	Level 3	3.1	1.2	5.4	1.9
	*p*-value	0.003	<0.001	0.019	0.002
Smoking	Non-smoker	7.6	11.3.	9.8	2.5
	Smoker	10.1	20.2	13.8	8.7
	*p*-value	0.039	<0.001	<0.001	<0.001
BMI	BMI <24	5.7	0.6	11.9	2.5
	24 ≤ BMI <28	15.3	4.1	14.7	8.9
	BMI ≥ 28	21.2	6.9	18.6	11.1
	*p*-value	<0.001	<0.001	<0.001	<0.001
Alcohol consumption	Non-drinker	10.9	4.9	5.9	8.2
	Drinker	14.9	11.3	12.6	8.7
	*p*-value	0.014	<0.001	<0.001	0.131
Occupation	Manual work	7.7	7.9	9.4	2.5
	Intellectual work	12.1	13.2	9.1	6.9
	*p*-value	<0.001	<0.001	0.822	<0.001
Education level	Primary	13.1	1.5	19.8	6.7
	Secondary	10.9	0.8	19.7	5.9
	High school	8.7	1.4	20.1	3.7
	University and higher	5.9	0.9	19.5	4.1
	*p*-value	<0.001	0.451	0.585	0.778

*BMI, body mass index; TC, Total Cholesterol; TG, triglyceride; LDL-c, low-density lipoprotein cholesterol; HDL-C, high-density lipoprotein cholesterol*.

### Dyslipidemia and Correlated Factors

The multivariable logistic regression suggested that dyslipidemia was significantly associated with an older age (OR: 1.94, 95% CI: 1.49–2.32) and male sex (OR: 1.85, 95% CI: 1.39–2.21). The participants who smoked one cigarette per day were more likely to have dyslipidemia (OR: 2.02, 95% CI: 1.98–2.13). Moreover, the participants who consumed alcohol were more likely to have dyslipidemia than those who did not consume alcohol (OR: 1.33, 95% CI: 1.16–1.63). The results illustrated that overweight (OR: 1.23, 95% CI: 1.19–1.27) and intellectual workers (OR: 1.36, 95% CI: 1.11–1.72) were associated with dyslipidemia in all subjects (Table 3).

**Table 3:** Multivariate logistic regression analyses of dyslipidemia and associated factors.

**Variables**		**OR**	**95%CI**	***P***
Age	18–24	1		
	25–34	1.21	1.09–1.47	<0.001
	35–45	1.94	1.49–2.32	<0.001
Gender	Women	1		
	Men	1.85	1.39–2.21	0.001
Residence	Urban	1		
	Rural	0.99	0.73–1.120	NS
Overweight or obesity	No	1		
	Yes	2.013	1.791–2.412	<0.001
Drinking	No	1		
	Yes	1.33	1.16–1.63	0.000
Smoking	No	1		
	Yes	2.02	1.98–2.13	<0.001
Occupation	Manual work	1		
	Intellectual work	1.36	1.11–1.72	0.003
Education level	Primary	1		
	Secondary	0.79	0.63–0.99	0.056
	High school	0.80	0.58–1.01	0.059
	University	0.93	0.66–1.32	0.457
Physical activity	Level 1	1		
	Level 2	0.89	0.46–1.77	NS
	Level 3	0.59	0.33–0.96	NS
Diabetes mellitus	No	1		
	Yes	1.17	0.89–1.75	NS
Hypertension	No	1		
	Yes	1.20	0.92–1.78	NS

*NS, P > 0.05*.

## Discussion

The increasing prevalence of stroke in young adults has become a worldwide public health problem. The prevalence varies widely according to socioeconomic, cultural, and ethnic factors. In our cross-sectional epidemiological study, we revealed a high prevalence of dyslipidemia among adults aged between 18 and 45 years in the southeastern coastal areas of China. This result is similar to a finding reported in a previous study (13) but higher than the figures obtained 10 years ago in Mainland China (8) and other developing countries (14, 15). Therefore, the prevalence has increased. The two major types of dyslipidemia observed in the young adults were hypertriglyceridemia and low HDL-C, which is consistent with other studies conducted in Asian countries (15, 16). We presumably suggest that this phenomenon may be related to the Westernization of young people's lifestyles and habits, such as diet patterns.

In our present study, we found that the mean lipid levels increased with age. A previous study identified a correlation between age and cholesterol levels (17), and our results are consistent with the aforementioned report. The potential explanations for age-related disorders of lipoprotein metabolism in humans, mainly focus on changes in the liver sinusoidal endothelium, post-prandial lipemia, insulin resistance induced by free fatty acids (FFA), growth hormone, androgens, and the expression and activity of peroxisome proliferator-activated receptors (18). We observed that the prevalence of dyslipidemia in young males was higher than that in young females, and the concentration of blood lipids in young males was higher than that in young females. The male sex could be a risk factor for dyslipidemia. This phenomenon might be caused by excessive fat accumulation, which results from being under a considerable amount of pressure and lacking enough exercise, as men constitute the main labor force in society. Furthermore, male lifestyle choices, such as smoking and consuming alcohol, are associated with dyslipidemia, while estrogen has a certain protective effect on lipid levels (19). Our study found that young people with higher levels of education had lower rates of H-TC than those with lower education levels, but this finding does not apply to H-TG, L-HDL, or H-LDL. This finding is consistent with several other studies (20, 21). The higher the level of education, the more likely people are to pay attention to their health condition and take measures to control their blood lipids.

The results of this study further suggest that the rate of dyslipidemia in urban youth and rural youth is 33.9 and 34.5%, respectively. There was no significant difference in dyslipidemia between urban and rural areas, which differs from the results reported in a previous study (22). China has been recognized as having large disparities between urban and rural areas, including socioeconomic status (23), health care systems, diet patterns, and nutritional status (24). However, Wenzhou is a developed medium-sized coastal city in southeastern China. With the rapid development of the rural economy since the economic reformation, communication between urban and rural areas has become more frequent. The lifestyles and dietary patterns (e.g., protein) of urban and rural youth are gradually becoming more similar, which may explain the similar blood lipid findings between the urban and rural populations. Furthermore, fish are a common type of seafood consumed by local people, and fish represent a rich source of docosahexaenoic acid (DHA) and eicosapentaenoic acid (EPA). The high consumption of these fatty acids has been related to lower serum triglycerides and higher HDL-C (25, 26). Studies have also found that cod protein reduced LDL cholesterol in overweight healthy adults (27) and affected animals (28). Our study revealed that young people who exercise for long durations have a lower prevalence of dyslipidemia. A previous study revealed that exercise had an effect on lipid levels by reducing LDL levels (29). A 4-years follow-up observational study conducted in Japan found that body weight reduction could improve lipid levels in men. Improvements in physical activity have been associated with body weight reduction (30). Regular exercise is regarded as an important part of health optimization and longevity. Exercise uses lipids as a source of fuel and improves the work capacity of skeletal muscles, ultimately increasing the blood supply to different parts of the body. Dietary changes and weight loss can lower LDL cholesterol, while exercise and low-carbohydrate diets can lower serum triglycerides and reduce the risk of cardiovascular disease.

We observed an association between occupation and dyslipidemia. The prevalence of dyslipidemia was significantly higher among intellectual workers in terms of TC, LDL-C, and TG, but this relationship was not observed for HDL-C. Intellectual workers often experience a great amount of sedentary time at work, lack exercise, and eat high-calorie diets, such as burgers, fried foods, and takeout. Such workers even need to eat supper while working at night. One study confirmed that sedentary time was associated with hyperlipidemia (31). Moreover, current smoking was associated with an increased risk of dyslipidemia. Smoking has been clearly established to regulate the levels of plasma lipids. A previously proposed possible explanation suggests that nicotine stimulates the secretion of catecholamines, cortisol, and growth hormones in humans, leading to an increase in serum-free fatty acid concentration, further stimulating the secretion of very low-density lipoproteins and triglycerides in the liver (32). In addition, smoking can destroy lipoproteins and induce lipid peroxidation. In contrast, smoking cessation can reverse the effect of smoking on serum lipids/lipoproteins (33). Smoking in young people is becoming increasingly worse. A national survey showed that the prevalence was increasing among young adults (34). Urgent strategies are needed for the promotion of smoking cessation, especially among adolescents. The relationship between alcohol and plasma lipid levels has been studied for decades and is controversial (35). Low-dose alcohol consumption has been shown to increase levels of HDL cholesterol, apolipoprotein A–I, and adiponectin and decrease the level of LDL cholesterol. However, alcohol consumption has also been associated with high TC and high TG (36). Excessive alcohol intake damages cholesterol homeostasis and results in increased hepatic cholesterol production and TC levels (37). The duration of alcohol consumption is significantly associated with hypercholesterolemia (38). This relationship was explained by alcohol consumption inhibiting the oxidation of free fatty acids, resulting in an increase in TGs. Alcohol induces an increase in the HDL-C level, which is related to the selective enrichment of polyunsaturated fatty acids (39) or the inhibition of the enzymatic activity of the cholesteryl ester transfer protein (CETP) to transport cholesterol to LDL-C particles. In addition, studies have shown that alcohol exposure activates hepatic proprotein convertase subtilisin/kexin type 9 (PCSK9), possibly regulating low-density lipoprotein cholesterol (40, 41). Our study showed that alcohol consumption is a risk factor for dyslipidemia, which may be related to the frequency of alcohol consumption in young people and excessive drinking.

There are major and distinctive strengths in our study including the large sample size and highly standardized sampling method. However, some limitations should be noted. Firstly, our study was a population-based cross-sectional study with no strict follow-up design. The prevalence of dyslipidemia was based on questionnaires and measurements during a single visit, implying that our results could have been affected by recall bias and unmeasured confounding. Secondly, the definition of dyslipidemia in our study is based on the Chinese guidelines for the prevention and treatment of dyslipidemia in adults, and we should compare our results with those obtained in other countries. Further epidemiological studies are needed to obtain more comprehensive information for the development of prevention and control measures. Thirdly, the concentration of LDL-C was calculated using the Friedewald formula, which can be seriously affected by some clinical parameters (42).

In conclusion, our study found that the prevalence of dyslipidemia in the young population in Wenzhou is relatively high. Therefore, young people should increase their health-promoting behaviors and consider the factors influencing dyslipidemia to lower the prevalence of dyslipidemia. It is crucial for the government to pay more attention to preventing and managing lipid disorders in this area. Accordingly, screening and controlling dyslipidemia in young adults constitutes a practical health strategy to prevent stroke in young people.

## Data Availability Statement

All datasets generated for this study are included in the article/supplementary material.

## Ethics Statement

The studies involving human participants were reviewed and approved by Institutional Review Board and Ethics Committee of The Second Affiliated Hospital of Wenzhou Medical University. The patients/participants provided their written informed consent to participate in this study.

## Author Contributions

HZ and K-YC were the main authors and assisted with the questionnaire development and distribution as well as data collation and analysis. WK and J-YY: formal analysis. X-DL and M-MS: investigation. X-DL, M-MS, and K-YC: data curation. HZ: writing—original draft and writing—review and editing. B-BC and K-YC: supervision. All authors contributed to the article and approved the submitted version.

## Conflict of Interest

The authors declare that the research was conducted in the absence of any commercial or financial relationships that could be construed as a potential conflict of interest.

## Abbreviations

HDL-C: high-density lipoprotein cholesterol
LDL-C: low-density lipoprotein cholesterol
TGs: triglycerides
TC: total cholesterol
OR, odds ratio, CI: confidence interval
BMI: body mass index
SBP: systolic blood pressure
DBP: diastolic blood pressure
UA: blood uric acid
FPG: fasting plasma glucose
FFA: free fatty acids
DHA: docosahexaenoic acid
EPA: eicosapentaenoic acid
CETP: cholesteryl ester transfer protein
PCSK9: proprotein convertase subtilisin/kexin type 9.
